# Clinical Implication of the Acumen Hypotension Prediction Index for Reducing Intraoperative Haemorrhage in Patients Undergoing Lumbar Spinal Fusion Surgery: A Prospective Randomised Controlled Single-Blinded Trial

**DOI:** 10.3390/jcm11164646

**Published:** 2022-08-09

**Authors:** Jung Min Koo, Hoon Choi, Wonjung Hwang, Sang Hyun Hong, Sang-Il Kim, Young-Hoon Kim, Seungtae Choi, Chang Jae Kim, Min Suk Chae

**Affiliations:** 1 Department of Anaesthesiology and Pain Medicine, Seoul St. Mary’s Hospital, College of Medicine, The Catholic University of Korea, 222 Banpo-daero, Seocho-gu, Seoul 06591, Korea; 2 Department of Orthopaedics, Seoul St. Mary’s Hospital, College of Medicine, The Catholic University of Korea, Seoul 06591, Korea; 3 Department of Anaesthesiology and Pain Medicine, St. Vincent’s Hospital, College of Medicine, The Catholic University of Korea, Seoul 06591, Korea; 4 Department of Anaesthesiology and Pain Medicine, Eunpyeong St. Mary’s Hospital, College of Medicine, The Catholic University of Korea, Seoul 06591, Korea

**Keywords:** haemorrhage, Hypotension Prediction Index, induced hypotension, machine learning, lumbar spinal fusion

## Abstract

We investigated the clinical implication of the Hypotension Prediction Index (HPI) in decreasing amount of surgical haemorrhage and requirements of blood transfusion compared to the conventional method (with vs. without HPI monitoring). A prospective, randomised controlled-trial of 19- to 73-year-old patients (n = 76) undergoing elective lumbar spinal fusion surgery was performed. According to the exclusion criteria, the patients were divided into the non-HPI (n = 33) and HPI (n = 35) groups. The targeted-induced hypotension systolic blood pressure was 80–100 mmHg (in both groups), with HPI > 85 (in the HPI group). Intraoperative bleeding was lower in the HPI group (299.3 ± 219.8 mL) than in the non-HPI group (532 ± 232.68 mL) (*p* = 0.001). The non-HPI group had a lower level of haemoglobin at the end of the surgery with a larger decline in levels. The incidence of postoperative transfusion of red blood cells was higher in the non-HPI group than in the HPI group (9 (27.3%) vs. 1 (2.9%)). The use of HPI monitoring may play a role in providing timely haemodynamic information that leads to improving the quality of induced hypotension care and to ameliorate intraoperative surgical blood loss and postoperative demand for blood transfusion in patients undergoing lumbar fusion surgery.

## 1. Introduction

Induced hypotension is an anaesthetic technique intended to reduce surgical blood loss and aid visibility in the surgical field. Surgical patients under general anaesthesia have many potential sources of hypotension, including volatile anaesthetic, narcotics, and analgesics, such as opioids. Surgical bleeding and shifts in intravenous fluid resulting in third spacing also lead to intraoperative hypotension. One of the anaesthesiologists’ roles is to maintain proper induced hypotensive care under the guidance of haemodynamic parameters. Severe blood pressure fluctuations can lead to catastrophic complications, such as cardiac arrest, neurological deficit, myocardial ischaemia or infarct, acute kidney injury, and unilateral or complete visual loss, particularly during spine surgeries in the prone position [1,2]. Due to the fatal complications of intraoperative hypotension, various monitors are used to avoid excessive fluctuations in vital signs.

The Hypotension Prediction Index (HPI) is an algorithm developed by Edwards Lifesciences (Irvine, CA, USA) for the EV 1000 system. The HPI uses features from the arterial waveform, such as time, amplitude, and slope, to predict hypotension, defined as systolic blood pressure (SBP) of 80–90 mmHg, mean arterial pressure (MAP) of 50–65 mmHg for more than 1 min, or a 30% decrease in the baseline MAP [3]. The HPI predicts hypotension 5 min in advance with 92% sensitivity and specificity and predicts hypotension 10 min in advance with 89% sensitivity and 90% specificity [4]. It still has 75% sensitivity and specificity for predicting hypotension 15 min in advance [5]. The index ranges from 0 to 100, and the higher the number, the more likely it is that the patient is hypotensive. The machine is designed to alert the physician with an audible alarm sound if the HPI is >85. Due to its real-time display and good predictability of impending hypotension, it is used in major abdominal, vascular, coronary revascularization surgery; on-pump cardiac surgery; and hip surgery to prevent hypotension [6,7]. The superiority of the HPI compared to static haemodynamic parameters has been proven in terms of decreasing the incidence and duration of hypotension intraoperatively and postoperatively [8,9]. The index has also been used in intensive care units to successfully predict hypotension in mechanically ventilated COVID-19 patients [5].

This study compared the volume of intraoperative blood loss and total requirement of blood products during lumbar spinal fusion surgery in patients under induced hypotension with conventional arterial blood pressure monitoring to those under HPI guidance for maintaining intraoperative hypotension.

## 2. Patients and Methods

### 2.1. Ethical Considerations

This was a prospective randomised, controlled, single-blinded trial in patients undergoing elective lumbar spinal fusion surgery. This study was approved by the institutional review board of Catholic University of Korea, St. Mary’s Hospital (KC21DISS0214) on 28 June 2021, and conformed with the Declaration of Helsinki. The study protocol was registered at the clinical research information service (CRIS) (KCT0006350) on 16 July 2021. Informed consent was acquired from the patients on the day before the surgery. The date of the first patient enrolled was 1 September 2021 and the data collection began on that day, until the end of the data acquisition of the last patient on 30 November 2021.

### 2.2. Study Population

Patients admitted to the Orthopaedic Surgery Department of Spinal Surgery, Catholic University of Korea, St. Mary’s Hospital, and who were scheduled for elective lumbar spinal fusion surgery were enrolled. Patients between 19 and 73 years old with an American Society of Anaesthesiology (ASA) classification I or II were enrolled. Exclusion criteria included those who refused to be part of the study, those with ASA classification of III or more, preoperative anaemia (haemoglobin level < 13.0 g dL^−1^ in males and <12.0 g dL^−1^ in females), or preoperative hypotension (mean blood pressure < 65 mmHg) [10]. Patients with uncontrolled resistant hypertension (blood pressure > 140/90 mmHg and those taking three or more antihypertensive medications) were also excluded [11]. Patients having surgery due to traumatic fracture of lumbar vertebrae or those with cerebrovascular diseases, such as coronary artery disease, congestive heart failure, arrhythmia, or previous cerebral infarction, were excluded. Patients with renal dysfunction and an estimated glomerular filtration rate < 60 mL min^−1^ 1.73^−3^, previous renal parenchymal disease, or those undergoing dialysis were excluded because of the possibility of a postoperative renal infarct. Those with preoperative coagulopathy with an INR > 1.5 or a platelet count < 100 × 10^9^ L^−1^ were also excluded.

**Figure 1:**
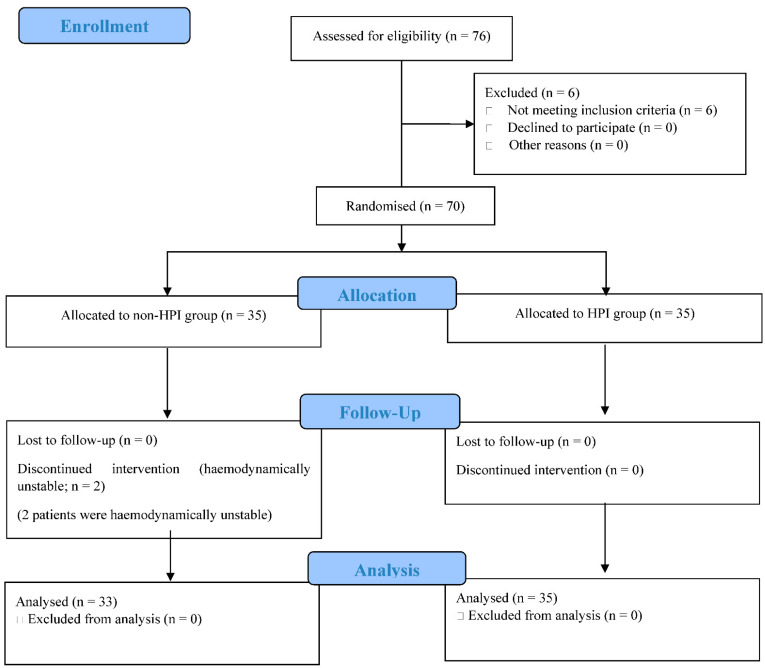
CONSORT flow diagram showing each stage of the randomised controlled trial; enrolment, allocation, follow-up, and analysis.

A total of 76 patients were included in the study. Of these, 68 patients were enrolled for data evaluation; 33 patients were placed in the non-HPI group, and 35 patients were placed in the HPI group. Six patients were excluded because they had coronary artery or cerebrovascular disease and two patients from the non-HPI group were excluded because they were hypertensive in the operating room beyond the inclusion criteria (Figure 1).

### 2.3. Randomisation

The patients were randomly assigned to the HPI or non-HPI groups, and they were blinded as to which group they had been allocated to. Randomisation was performed using sealed, opaque envelopes containing the group assignments generated by a computer tool (1:1 ratio) to ensure an equal distribution of treatment assignments across the entire study period. When an enrolled patient arrived in the holding area, the topmost envelope was opened by the attending anaesthesiologist. The attending anaesthesiologist and nurses in the operating room, who were not involved in further patient care or data collection (other than filling in medical record forms), were aware of the group allocations.

### 2.4. Posterior Lumbar Interbody Fusion and General Anaesthesia

The lumbar interbody fusion surgeries were completed by an experienced orthopaedic team (S.I.K. and Y.H.K.) [12]. Briefly, the patients were placed in the prone position without abdominal pressure to decompress the epidural venous plexus and decrease intraoperative haemorrhage. An open midline approach was applied to subperiosteally expose the spinous process, laminae, facets, and transverse process of the involved anatomic parts. The pedicle screws were inserted before or after a laminectomy. Bony resection was performed laterally to set the complete or partial excision of the superior and inferior articular processes. The facets were usually excised completely to reconstruct deformity. Annulotomy, discectomy, and endplate preparation were serially presented. The cage or bone graft was placed into the interspace through each of the two annulotomy procedures. Supplemental pedicles and rod constructs were placed. Surgical drainage was inserted after the fusion of two or more vertebrae levels.

Balanced general anaesthesia was performed by attending anaesthesiologists and nurses who were aware of the allocated group but were not involved in further outcome measures. All patients had their radial arteries cannulated for continuous blood pressure monitoring, which was connected to the HemoSphere monitor platform (Edwards Lifescience, Irvine, CA, USA). At least 7 mL kg^−1^ h^−1^ crystalloid was infused as maintenance fluid, and intraoperative blood loss was supported by further crystalloid infusion of up to three-fold of blood loss or the equivalent amount of colloids. Red blood cells were transfused when ongoing blood loss resulted in intraoperative haemoglobin level < 7.0 g dL^−1^ [13].

### 2.5. Induced Hypotensive Anaesthesia and HPI Monitoring

The induced hypotension technique was carried out with the targeted SBP of >100 to ≥80 mmHg or a decrease of 30% in preoperative SBP. Induced hypotension was initiated from the point of perivertebral muscle retraction to the end of the surgery before the skin was sutured. Nicardipine HCl (5 mg mL^−1^, dihydropyridine, class of calcium channel blocker, BC World Pharm., Seoul, Republic of Korea) was the drug of choice to induce and maintain hypotension. Nicardipine (20 mg) was mixed with normal saline to a total of 40 mL solution. The nicardipine solution was continuously infused through a Terufusion TE-311 syringe pump (Terumo Corp., Hatagaya Shibuya-ku, Tokyo, Japan), at a rate of 5–15 mL h^−1^. The rate of infusion was increased by 5 mL h^−1^ or bolus loading of 1 mL was applied to reach the targeted SBP. Repeated bolus doses of nicardipine were allowed by the attending anaesthesiologists’ judgement to reach the targeted study plan of SBP. However, after the modification of infusion rate or bolus infusion, additional adjustment was pended until the onset of action of nicardipine, which was 1 to 2 min. When SBP was <80 mmHg or more than 30% compared to the preoperative SBP, 5–10 mg ephedrine was administered as a rescue drug. SBP was targeted at the same level (80–100 mmHg) in the HPI group. However, further efforts were given to maintain the HPI > 85. Incremental increases of 5 mL h^−1^ or bolus loading of 1 mL nicardipine were applied to maintain the HPI at 85–100. However, in order to avoid serious complications related to intraoperative hypotension, rescue drugs were given to raise blood pressure if SBP dropped more than 30% of the baseline, even if HPI was <85.

Identical, single HemoSphere monitors were used to display the parameters of the two groups. However, a FloTrac sensor was attached to the arterial catheter in the control group and the non-HPI group. Haemodynamic information on the patients’ cardiac output, cardiac index, stroke volume, and variation in stroke volume were displayed on the HemoSphere monitor. The Acumen IQ sensor was connected to the radial arterial line. The HPI was also displayed on the HemoSphere monitor.

### 2.6. Outcome Measurements

The primary outcome was the volume of intraoperative surgical blood loss. The volume of blood that drained into the suction bottles was calculated by subtracting the volume of irrigated saline from the total volume of fluid and the blood mixture in the suction bottle. Blood smudged to the surgical gauze was counted using visual guidance by estimating the percentage of blood saturation [14]. Intraoperative blood volume was determined at the end of the surgery.

The secondary outcome included the haemoglobin level. Preoperative haemoglobin levels (pre-Hb), initial haemoglobin after inducing anaesthesia (T0), at the end of surgery (T1), and haemoglobin levels measured on postoperative days (POD) 1, 2, and 3 after surgery were recorded. The initial haemoglobin level (T0) was measured from the patient’s arterial catheter after catheterisation before the patient was placed in the prone position. T1 was measured at the end of surgery when the patient was switched to the supine position, before removing the endotracheal tube. Haemoglobin levels at the specified time points were compared between the non-HPI and HPI groups. Changes in levels between (1) T0 and T1, (2) T0 and POD 1, (3) T0 and POD 2, and (4) T0 and POD 3 were recorded. The incidence of transfusion during and after surgery was recorded.

### 2.7. Clinical Variables

The preoperative findings included sex, age, body mass index, blood pressure, heart rate, ASA physical classification, comorbidities, and laboratory variables. Intraoperative findings included operation time, operation site, infusion drugs, and fluid and blood transfusions. Postoperative complications were assessed using the Clavien-Dindo classification [15].

### 2.8. Statistical Analysis

The minimum sample size required was determined based on that needed to detect a difference in the total amount of intraoperative haemorrhage between patients with/without HPI monitoring. Based on a preliminary study conducted at our hospital (unpublished), mean intraoperative haemorrhage in the HPI group and the non-HPI group were 398 mL and 512 mL, respectively, and the standard deviation (SD) was 161 mL. Therefore, a minimum sample size of 32 patients in each group was required (α = 0.05, power = 0.8). We recruited 35 patients into each group, assuming a dropout rate of 10%.

The normality of the distribution of continuous data was evaluated using the Shapiro–Wilk test. Continuous data are presented as mean and standard deviation. Categorical data are presented as absolute numbers and percentages, as appropriate. Continuous data between the groups were compared using a Student’s *t*-test, and serial changes in each group were analysed using the paired *t*-test. Categorical data were analysed with the *χ*^2^ test or Fisher’s exact test, as appropriate; *p*-values < 0.05 were considered significant. All analyses were performed with SPSS software for Windows (version 26; IBM Corp., Armonk, NY, USA).

## 3. Results

### 3.1. Pre- and Intraoperative Clinical Findings

A total of 68 patients were enrolled for data evaluation. The mean age of the patients was 64 ± 7.9 years, with median (IQR) of 66 (61–69) years old. Nearly all patients had their lumbar vertebrae fusion surgery because of spinal stenosis. A single patient, a 35-year-old man and the youngest of all, had disc herniation requiring surgical fusion. The demographic and intraoperative findings were comparable between the two groups (Table 1 and Table 2).

### 3.2. Intraoperative Haemorrhage Outcomes

**Figure 2:**
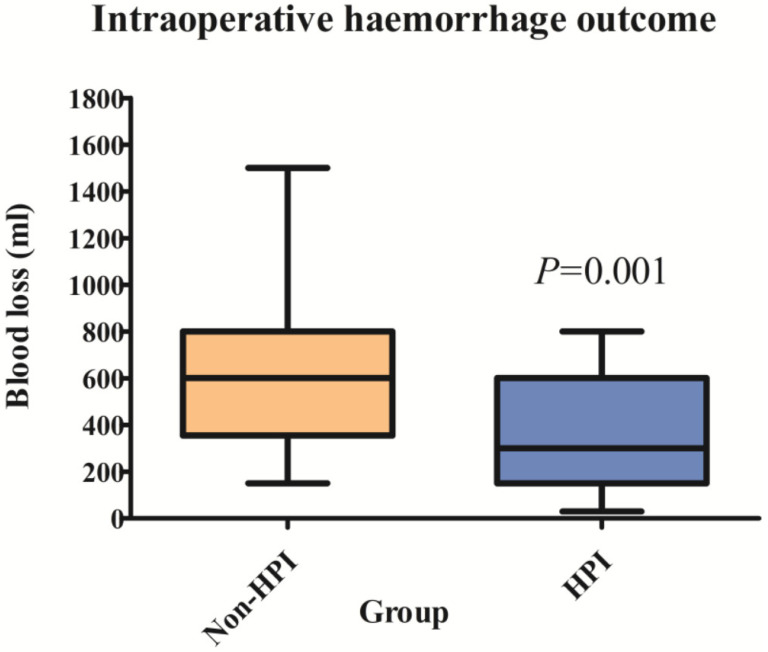
Intraoperative haemorrhage outcome comparing the amount of intraoperative surgical bleeding between the non-HPI group (orange box) and the HPI group (blue box). The mean ± SD intraoperative bleeding was 532 ± 232.7 mL, while the amount of surgical bleeding was 299.3 ± 219.8 mL with a *p*-value of <0.001.

Intraoperative blood loss was significantly lower in the HPI group than in the non-HPI group (Figure 2). Blood loss differed at surgical spinal level 1–2 between the groups (Table 3).

### 3.3. Perioperative Haemoglobin and Blood Transfusion Outcomes

The haemoglobin level at the beginning and end of surgery did not differ significantly between the two groups. However, a difference was observed on POD 1, being higher in the HPI group. The change in level (vs. T0) was larger in the non-HPI group, and the results were significant on PODs 1 and 2 (Table 4). The postoperative requirement for a red blood cell transfusion was much higher in the non-HPI group (Table 5).

### 3.4. Postoperative Clinical Outcomes

All patients were alert after the surgery without any neurological deficits. No other serious postoperative complications occurred, as defined by the Clavien–Dindo classification of surgical complications. All patients had none to minor complications of class I or II, according to the Clavien–Dindo classification.

## 4. Discussion

The result of our study showed that the HPI group had decreased surgical blood loss in lumbar spinal surgery. Spinal fusion surgery is a major surgery that results in a large amount of bone and tissue bleeding. Although bleeding may vary according to the surgical scope, blood loss ranges from 100 to 3100 mL in non-instrumented spinal fusion and 360 to 7000 mL in instrumented fusion [16]. Patients undergoing spinal fusion surgery are mostly older, which is associated with an increased risk of intraoperative bleeding [17]. Massive intraoperative haemorrhage can lead to an allogenic blood transfusion with high 30-day mortality [18] and accompanying serious postoperative complications, such as postoperative ventilator care for more than 48 h, sepsis, pneumonia, return to the operating room, and transfusion-related infections [19].

Several modalities are available to reduce intraoperative blood loss such as controlled hypotension [20]. Pharmacological interventions include inhalation anaesthetics, calcium channel antagonists, sodium nitroprusside, nitroglycerin, remifentanil, and milrinone [3,21]. Of these, nicardipine is a dihydropyridine calcium channel blocker that lowers blood pressure via a vasodilatory effect. It has no chronotropic, dromotropic, or inotropic effects, and its use is favoured in controlled hypotension because of its rapid onset and offset. It has been used in various surgeries, including total hip surgery, orthognathic surgery, and spinal surgery in adults and children [22,23,24,25]. However, understanding the safe adequate level of hypotension is challenging. “Normal” blood pressure in terms of cerebral autoregulation differs among patients due to differences in arterial tension. Cerebral ischemia due to hypotension develops in the Circle of Willis at a MAP of 40–55 mmHg in a normotensive adult positioned in a vertical posture, and at 45–55 mmHg in the supine position [26]. However, different thresholds apply to hypertensive patients, so an advanced haemodynamic monitoring device may be required to improve their haemorrhage outcomes without adverse events.

Machine learning-guided intraoperatively controlled hypotension anaesthesia may help decrease the volume of surgical blood loss compared to conventional monitoring-based anaesthesia during spinal fusion surgery. The HPI is an emerging vital sign parameter that was developed to predict hypotension in advance. It provides an early-warning system and therefore decreases the number of intraoperative hypotensive events [27]. It predicts hypotension 15 min in advance and is faster than cardiac output, stroke volume, MAP, pulse pressure, heart rate, stroke volume variation, and the shock index [8]. The benefit of the HPI in major cardiac, thoracic, and vascular surgery has been demonstrated, where maintaining intraoperative haemodynamic stability is important [28]. Despite its initial purpose, we utilised the HPI to intentionally induce intraoperative hypotension, not to prevent hypotension. By simply adding a stopcock to the arterial blood cannula, the HPI was easily monitored with good sensitivity and specificity [4]. We presumed that HPI-guided control of blood pressure would be more reliable than instantaneous arterial blood pressure because systemic hypotension can be predicted and maintained in advance. Furthermore, we presumed that HPI-guided induced hypotension would be more customised to each patient who has a different auto-regulatory mechanism. To the best of our knowledge, this is the first study to utilise the HPI to induce hypotension. HPI-guided controlled hypotension decreased intraoperative blood loss by about 230 mL compared to the non-HPI group. About 220 mL bleeding was avoided in patients undergoing lumbar vertebrae fusion in the HPI group.

One of our concerns was that by adding the HPI monitor, the HPI group might require more fluid, nicardipine, and vasoactive drugs intraoperatively. Interestingly, neither ephedrine, nicardipine, nor fluid administration to resuscitate hypotension differed between the two groups. Similar results were reported by a study comparing time-weighted hypotension in HPI and control groups during non-cardiac surgery [29]. We suggest that HPI-guided anaesthetic hypotension can decrease intraoperative blood loss without overdosing on hypotensive medications. In addition, intraoperative blood loss is related to the deterioration of haemoglobin level. HPI-guided controlled hypotension led to smaller decreases in the haemoglobin level on POD 1. Smaller changes in the postoperative haemoglobin level from the baseline were observed on POD 1 and POD 3 in the HPI group. The postoperative requirement for a red blood cell transfusion was higher in the non-HPI group. HPI-guided controlled intraoperative hypotension was successfully carried out without any postoperative complications. HPI-guided controlled intraoperative hypotension was successfully carried out without any postoperative complications (Clavien–Dindo classification ≤ II).

Our study had several limitations. First, the design of the study was not completely blinded, due to the nature of the monitor. The HPI group had the HPI numbers shown to the anaesthesiologists. Second, the definition of intraoperatively SBP oriented induced hypotension of 80–100 mmHg was targeted identically between the patients with and without hypertension and from young (35 years old) to old ages (73 years). Flawless modulation of hypotension is very difficult because “normotension” differs among patients. More than half of the study population had hypertension as a preoperative comorbidity, and it was inevitable that most of the surgery population was older because the mean age of the whole study population was 64. Further studies are required to explain the efficacy and safety of the HPI according to variety of ages and degrees of baseline blood pressure. Additrionally, the duration of hypotensive time, or the number of hypotensive episodes, were not recorded. Although we administered hypertensive drug ephedrine as soon as SBP reached < 80 mmHg or dropped more than 30% compared to the preoperative SBP, severe hypotension in longer durations may lead to postoperative complications. Third, although there were no differences in the amount of intraoperative nicardipine and ephedrine used, the timing of when the drugs were administered was not compared between the two groups. Therefore, the quality of HPI-guided hypotension in terms of how fast the physician can control the blood pressure of the patients was not shown. Fourth, this was a single-centre study with a small study population. Small sample size may limit the generalizability of the study to other surgery or general population. More studies with a larger population may be needed to demonstrate the beneficial effects of HPI for controlled hypotension. Further studies may demonstrate the advantages of HPI-guided controlled hypotension, including preventing excessive use of hypotensive drugs with an early-warning system to anaesthesiologists, swift calibration of anaesthetic drugs, avoidance of unnecessary complications by putting patients at a hypotensive risk, and potential complications. Whether the range of HPI 85–100 is a generalizable target to all population is also questionable and needs further study for validation.

## 5. Conclusions

We explored the clinical implication of the HPI in induced hypotension during lumbar spinal fusion surgery. The use of HPI monitoring may play a role in providing timely haemodynamic information that leads to the improvement of the quality of induced hypotension care, amelioration of intraoperative surgical blood loss, and a decrease in the postoperative demand for blood transfusion in patients undergoing lumbar fusion surgery. Including a machine-learning-based HPI during conventional vital monitoring may be available to efficiently and safely manage the haemodynamic alterations without excessive burden of vaso-pressor or -dilator infusion.

## Figures and Tables

**Table 1:** Demographic findings.

Group	Non-HPI	HPI	*p* Value
n	33	35	
Sex (Female)	17 (51.5%)	24 (68.6%)	0.16
Age (years)	63 ± 8.7	64 ± 7.3	0.56
BMI (kg m^−1^)	26.1 ± 2.8	24.3 ± 3.2	>0.89
***Preoperative vital signs***			
SBP (mmHg)	134 ± 23	132 ± 20	0.71
DBP (mmHg)	79 ± 12	76 ± 9	0.42
MBP (mmHg)	97 ± 14	95 ± 12	0.52
HR (beats/min)	71 ± 10	73 ± 13	0.38
***ASA classifications***			
ASA I	2 (6.1%)	9 (22%)	0.1
ASA II	31 (93.9%)	26 (74.3%)	0.08
***Comorbidities***			
Diabetes mellitus	6 (17.9%)	8 (22.9%)	0.63
Hypertension	19 (54.1%)	19 (54.3%)	0.79
***Preoperative lab findings***			
WBC count (10^9^ L^−1^)	6.3 ± 1.9	6.7 ± 2.5	0.45
Neutrophil (%)	57.9 ± 9.1	57.2 ± 9.6	0.58
Lymphocyte (%)	30.8 ± 9.0	33.2 ± 9.0	0.23
Hb (g/dL)	13.1 ± 1.8	13.0 ± 1.3	0.74
Platelet count (10^9^ L^−1^)	235.7 ± 43.9	250.1 ± 60.6	0.24
INR	1.0 ± 0.1	1.1 ± 0.1	0.7
aPTT (s)	25.7 ± 2.0	25.7 ± 2.5	0.6
Glucose (mg dL^−1^)	108.6 ± 23.2	108.7 ± 26.7	0.7
Creatinine (mg dL^−1^)	0.73 ± 0.2	0.66 ± 0.2	0.12
Albumin (g dL^−1^)	4.2 ± 0.5	4.4 ± 0.4	0.06
AST (U L^−1^)	24.9 ± 9.5	23.8 ± 8.5	0.59
ALT (U L^−1^)	23.6 ± 13.3	23.6 ± 17.5	>0.999
Calcium (mg dL^−1^)	9.2 ± 0.5	9.3 ± 0.42	0.45
Sodium (mmol L^−1^)	139.3 ± 3.0	140.5 ± 2.4	0.55
Potassium (mmol L^−1^)	4.3 ± 0.3	4.3 ± 0.4	0.4
Chloride (mmol L^−1^)	103.5 ± 2.5	100.7 ± 15.8	0.3

**Abbreviations:** HPI, hypotension predictive index; BMI, body mass index; ASA, American Society of Anaesthesiology; WBC, white blood cell count; Hb, haemoglobin; INR, international normalised ratio; aPTT, activated partial thromboplastin clotting time; AST, aspartate aminotransferase; ALT, alanine aminotransferase. Values are expressed as mean ± SD and numbers (proportions).

**Table 2:** Intraoperative findings.

Group	Non-HPI	HPI	*p* Value
n	33	35	
***Operation time* (min)**	208 ± 59.3	207.9 ± 71.3	0.73
***Operation site***			
Involved level			
Levels 1–2	25 (75.8%)	27 (77.1%)	0.28
Levels 3–4	8 (24.2%)	8 (22.9%)	
***Infusion drug dose***			
Nicardipine (mg)	11.3 ± 7.2	12.9 ± 8.6	0.68
Ephedrine (mg)	10.7 ± 6.2	11.2 ± 11.6	0.43
Remifentanil (µg)	664.0 ± 686.4	492.4 ± 533.5	0.21
***Fluid and blood management***			
Hourly crystalloid infusion (mL kg^−1^ h^−1^)	7.3 ± 3.4	8.7 ± 4.2	0.1
Colloid requirement (%)	13 (39%)	13 (37.1%)	0.19
Hourly urine output (mL kg ^−1^ h^−1^)	1.4 ± 1.5	1.3 ± 1.0	0.41

**Abbreviation:** HPI, hypotension predictive index. Values are expressed as mean ± SD and number (proportion).

**Table 3:** Intraoperative haemorrhage outcomes by surgical level.

Group	Non-HPI	HPI	*p* Value
n	33	35	
***By surgical level* (mL)**			
Levels 1–2	513.6 ± 216	319.6 ± 226.1	0.003
Levels 3–4	930.0 ± 312.2	456.3 ± 255.6	0.005

**Abbreviation:** HPI, hypotension predictive index. Values are expressed as mean ± SD.

**Table 4:** Haemoglobin outcomes.

Group	Non-HPI	HPI	*p* Value
n	33	35	
***Haemoglobin level* (g dL^−1^)**			
T0	12.7 ± 1.6	12.4 ± 1.2	0.4
T1	11.3 ± 1.7 ^††^	11.5 ± 1.5 ^††^	0.63
POD 1	10.1 ± 1.7 ^††^	11.2 ± 1.5 ^††^	0.005
POD 2	10.3 ± 1.6 ^††^	10.9 ± 1.7 ^††^	0.13
POD 3	10.9 ± 1.6 ^††^	11.6 ± 1.4 ^†^	0.07
***Haemoglobin level difference* (g dL^−1^)**			
change in Hb (T0–T1)	1.3 ± 1.0	0.9 ± 0.8	0.023
change in Hb (T0–POD 1)	2.6 ± 1.5 ^††^	1.2 ± 1.8	0.001
change in Hb (T0–POD 2)	2.4 ± 1.3 ^††^	1.4 ± 2.0	0.029
change in Hb (T0–POD 3)	1.7 ± 1.8	0.8 ± 1.7	0.026

**Abbreviations:** HPI, hypotension predictive index; T0, immediately after the induction of anaesthesia; T1, at the end of the surgery; POD, postoperative day; Hb, haemoglobin. ^†^ *p* ≤ 0.01, and ^††^ *p* ≤ 0.001 compared to initial levels (haemoglobin at T0 or change in Hb (T0–T1)). Values are expressed as mean ± SD.

**Table 5:** Blood transfusion outcomes.

Group	Non-HPI	HPI	*p* Value
n	33	35	
***Intraoperative transfusion***			
Requirement of PRBC (%)	8 (24.2%)	6 (17.1%)	0.82
Requirement of FFP (%)	1 (3.0%)	1 (2.8%)	>0.999
***Postoperative transfusion* (*between PODs 1 and 3*)**			
Requirement of PRBC (%)	9 (27.3%)	1 (2.9%)	0.021
Requirement of FFP (%)	0 (0%)	0 (0%)	-

**Abbreviations:** HPI, hypotension predictive index; PRBC, packed red blood cells; FFP, fresh frozen plasma; POD, postoperative day. Values are expressed as numbers and proportions.

## Data Availability

The datasets generated and/or analysed during the current study are not publicly available because disclosing patients’ personal information is against the law, but de-identified datasets are available from the corresponding author on reasonable request.
